# Legal responses to Japan’s Fukushima Nuclear Wastewater Discharge into the sea—from the perspective of China’s right-safeguarding strategies

**DOI:** 10.1016/j.heliyon.2023.e15701

**Published:** 2023-04-25

**Authors:** Meng Li, Xuedong Wang

**Affiliations:** a School of Humanities and Law, China University of Petroleum (East China), Qingdao, 266580, China; b School of Humanities and Law, China University of Petroleum (East China), Qingdao, 266580, China

**Keywords:** Fukushima nuclear wastewater discharge, Illegality, Legal basis, China's right safeguarding strategies, International dispute settlement mechanisms

## Abstract

Since Japan announced its plan to discharge Fukushima nuclear wastewater into the sea on April 13, 2021, the discussions on the hazards and illegality of this act have never stopped. In this discharge crisis, countries neighboring Japan are direct stakeholders, so the coping strategies they will adopt particularly attract worldwide attention. The paper focuses on the Challenges of Fukushima Nuclear Wastewater Discharge into the sea and discussing China's responses to Fukushima nuclear wastewater discharge into the sea from the perspective of China's right-safeguarding strategies. It has reached the following conclusions: once Fukushima nuclear wastewater is discharged into the sea, it will result in a serious hazard and cause social and economic impacts on all aspects; the Japanese government's decision and act to discharge Fukushima nuclear wastewater into the sea are against laws; the legal basis for China to safeguard its rights includes China's domestic laws and Japan's domestic and international laws. Regarding the right-safeguarding strategy, China can act on the domestic and international levels to defend its rights and interests and safeguard the security of the ocean environment and humans.

## 1 Introduction

On April 13, 2021, Japan announced it will release 1.25 million tons of treated wastewater contaminated by the wrecked Fukushima Daiichi Nuclear Power Plant into the Pacific Ocean [1]. Unlike neighboring countries, the United States supports Japan's decision. US Secretary of State Antony Blinkentweeted to appraise Japan for its transparent efforts to deal with wastewater from the Fukushima nuclear plant [2]. With the proceeding of Japan's nuclear wastewater discharge plan, the world's severest nuclear pollution is approaching. The waters near Fukushima Prefecture not only serve as the economic source that coastal citizens rely on for survival, but are also vital parts of the Pacific Ocean and even the worldwide ocean. Its extensive amount of radioactive materials will exert an inestimable impact on ocean creatures, the natural environment, and human health. The general principle of our law is that loss from accident must lie where it falls [3]. China, a country neighboring Japan and a representative country along the coast of the Pacific Ocean, proposed its strong dissent to this nuclear wastewater discharge. If Japan insists on putting its own interests above the public interest of the international community and insists on taking the dangerous step, it will surely pay the price for its irresponsible behavior and leave a stain in history [4]. Because of this, how China will affix the legal responsibility for Fukushima nuclear wastewater discharge into the sea attracts worldwide concerns.

## 2 The damages of Fukushima Nuclear Wastewater Discharge into the sea

### 2.1 Scientific understanding and risk assessment

Japan's discharge of Fukushima nuclear wastewater into the sea will have a profound and far-reaching impact on the marine environment and human health. Radioactive elements in nuclear wastewater cause long-term radiological hazards, which may induce diseases and genetic mutations. After the accident at the Fukushima Daiichi Nuclear Power Plant (NPP), radionuclides (such as 131I and 134Cs) from this source were detected in the atmosphere of most Chinese cities and provinces [5]. Also, the State Oceanic Administration of China monitored the radioactivity in the marine environment of the West Pacific waters east of Japan three months after the Fukushima Nuclear Power Plant's leakage. According to monitoring results, radionuclides were detected in seawater samples, and 94% of samples in the monitoring station contained 134Cs, which didn't exist in normal circumstances. Besides, the content of 137Cs in 71% of monitoring stations exceeded the background range of the Chinese sea area, and the content of 137Cs and 90Sr was 300 and 10 times that of the background range of the Chinese sea area [6].

The risk assessment is based on the above scientific evidence to make a suitable and sufficient assessment for the consequences of the Fukushima nuclear wastewater discharge, which is mainly made by professional scholars. However, once Fukushima nuclear wastewater is discharged into the sea, it will result in the following hazards: irreversible damage, multiple-faceted safety hazards, and worldwide consequences. Its risks can be evaluated and summarized as follows: 1) Severe damage to marine ecosystems and animal mutations; 2) Exceedances of radiation levels in fishery products, fruits and vegetables, rice, and even in many segments of cosmetics; 3) The health of people in all countries along the Pacific coast [7].

### 2.2 Expected social and economic impacts

Japan's discharge of Fukushima nuclear wastewater into the sea will cause social and economic impacts on all aspects, which are mainly reflected in infringements upon the right to a healthy environment, the right to development, and the property right. On October 8, 2021, the right to a healthy environment was officially recognized by the UN's Human Right Council as a basic human right [8]. Currently, more than 150 countries have issued laws to safeguard this right [8]. Fukushima nuclear wastewater contains several radioactive elements that may damage the environment and humans and gravely impact ocean resources. Undoubtedly, this discharge act will severely infringe upon the right to a healthy environment. The environment is an ecological system shared by all humans. Every subject of environmental legal relations equally enjoys the right to a healthy environment, and no subject can damage other subjects' right to a healthy environment. Although Japan had access to and utilized environmental resources, it discharged nuclear wastewater after the Fukushima Nuclear Power Plant accident and refused to fulfill the obligation to protect the healthy environment. Its act severely infringed on other countries' or regions' right to a healthy environment.

The right to development is a human right that recognizes every human right for constant improvement of well-being. The right was first recognized in 1981 in Article 22 of the African Charter on Human and Peoples' Rights as a definitive individual and collective right [9]. The right to development was subsequently proclaimed by the United Nations in 1986 in the “Declaration on the Right to Development,” which was adopted by the United Nations General Assembly resolution 41/128 [10]. Japan's discharge of Fukushima nuclear wastewater infringes on the right to development of every country that may be affected. Firstly, discharging nuclear wastewater into the sea will damage the right to development on the economic development level. It's because nuclear pollution will thwart neighboring countries' fisheries and limit the economic development of coastal areas, failing to guarantee the affected developing and developed countries enjoy the same right to environmental development. Secondly, discharging nuclear wastewater into the sea will damage the right to development on the level of sustainable development. Environmental protection is a critical component of a country's development, and the irreversibility of nuclear pollution will severely damage the virtuous cycle of environmental protection and economic development.

The property right is also basic human right. Judged by the economic impacts caused by the Chornobyl incident, the economic factors for Japan's discharge act include but are not limited to the ocean product industry, tourism, and the catering sector. Due to impaired functions of soils in coastal regions, citizens in polluted regions are forced to move to other places, which may waste more economic interests. A year after the Fukushima Nuclear Power Plant accident, the Japanese government announced a series of standards for compensating citizens in the evacuation area of Fukushima's No.1 Nuclear Power Plant. These standards stipulated the compensations for immovable properties, including land and houses, house assets such as furniture and electrical appliances, and losses from operations and salaries [11]. Furthermore, the Japanese government and Tokyo Electric Power successively launched many legal precedents concerning economic compensation for victims of the nuclear accident. For example, the Japanese Yokohama District Court judged that 152 plaintiffs had the right to claim economic compensation in 2019, including the compensation for economic losses of evacuating from Fukushima Prefecture to Ehime Prefecture [12]. The relief measures in Japan have fully demonstrated the Fukushima nuclear power plant incident's damage to citizens' right to property. Nevertheless, the Japanese government didn't respond to or formulate plans to compensate for the economic losses other countries and regions suffered due to the Fukushima Nuclear Power Plant accident and the discharge act.

## 3 Illegality of Fukushima Nuclear Wastewater Discharge into the sea

### 3.1 Illegality of Japan's decision-making procedures

Japan is a contracting party to the *United Nations Convention on the Law of the Sea (UNCLOS)*. According to Articles 192 and 194 in the *UNCLOS*, Japan has an obligation to protect and preserve the marine environment and shall take all measures consistent with this Convention to prevent, reduce, and control pollution. Besides, it shall take all measures to ensure domestic environmental pollution incidents do not cause damage by pollution to other states or spread beyond the areas where it exercises its sovereign rights. Considering the discharge of nuclear wastewater into the sea will increase the risk of polluting the marine environment, Japan should “observe, measure, evaluate and analyze, by recognized scientific methods, the risks or effects of pollution of the marine environment”, publish a report on environmental impact assessment, and notify the discharge plan and its environmental impact assessment report to IAEA (International Atomic Energy) and all countries that may be affected in advance [13]. In Pulp Mills on the River Uruguay Case,^1^ the International Court of Justice (ICJ) creatively proposed environmental impact assessments. The ICJ held that environmental impact assessments should be conducted if industrial activities have severe adverse impacts on the cross-border environment and resource sharing. Therefore, Japan should conduct adequate environmental impact assessments before discharging nuclear wastewater and report evaluation results to stakeholders. Nevertheless, Japan hasn't fulfilled the procedural obligations stipulated in the Convention. Since the Japanese government's decision-making procedures for discharging nuclear wastewater into the sea lack openness, transparency and democracy, the Japanese government is suspected of violating the procedural obligation stipulated by the *UNCLOS*.

### 3.2 Illegality of the act to discharge Fukushima nuclear wastewater into the sea

The illegality of Japan's act to discharge Fukushima nuclear wastewater into the sea can be analyzed from two perspectives. For one thing, Article 194 of the *UNCLOS* stipulates that Japan has an obligation to take all necessary measures to prevent, reduce, and control pollution in the marine environment. Whether there is a more proper alternative plan is the criterion for judging whether Japan “individually or jointly as appropriate, all measures consistent with UNCLOS”. There are five disposition paths considered by the Government (ground injection, controlled discharge into the sea, discharge as steam, discharge as hydrogen, and solidification for underground burial) [14]. In the Southern Bluefin Tuna Cases,^2^ the International Tribunal for the Law of the Sea (ITLOS), under the propositions of Australia and New Zealand (the applicants), ordered Japan to terminate its plan for the experimental catch of southern bluefin tunas. This case's significance is the Tribunal restricted cross-border fishery in the precautionary principle, which enhances the stipulations and spirit of the Rio Declaration 1992 to some extent.^3^ Regarding Japan's nuclear wastewater discharge, there is inadequate full scientific certainty to prove the hazards of discharging before and after the discharge act. Hence cost-effective measures can be taken to prevent environmental degradation.

However, the Japanese government insists on selecting the plan of discharging nuclear wastewater into the sea, which considerably increases the risks of severe damage to the global marine environment. It is sufficient to prove that the Japanese government didn't “take all measures consistent with UNCLOS” to avoid pollution to the marine environment. Therefore, the act to discharge Fukushima nuclear wastewater into the sea violates the *UNCLOS* and the basic legal principle of international radiation prevention. For another, the future act to discharge should meet existing standards for safe discharge. Currently, the international standards for the safe discharge of nuclear wastewater can be determined based on the *UNCLOS* and the *Convention on the Prevention of Marine Pollution by Dumping of Wastes and Other Matter 1972 (London Convention)*. According to Article 207 of the *UNCLOS*, “States shall adopt laws and regulations to prevent, reduce and control pollution of the marine environment from land-based sources … taking into account internationally agreed rules, standards and recommended practices and procedures.” This has been recognized by relevant international conventions. For example, the *London Convention* stipulates the wastes allowed to be dumped and their lowest exempted concentrations. Japan's future discharge of nuclear wastewater in the sea may result in two circumstances. Firstly, the activity concentration of radionuclides is higher than the safety discharge criteria stipulated by domestic laws and the IAEA, so it's inevitably against the laws. Secondly, the activity concentration of radionuclides is lower than relevant safe discharge criteria, and the Japanese government should confirm it by checking the oversight mechanism on the prerequisites of openness, transparency, and democracy. In addition, the Japanese government should timely update the implementation of the plan for discharging nuclear wastewater into the sea, the report on environmental impact assessment, and emergency preparation plans. Otherwise, it remains an unlawful act.

## 4 Legal basis for China to safeguard its rights

All parties in the international community should cooperate before damaging behaviors occur. It's an integral component of the principle for international cooperation, the precise application of the risk prevention principle, and the best annotation of protecting the ecological environment in the case of Japan's discharge of nuclear wastewater into the sea. The compensation and remedy for damage after the damaging act occurs are based on the legal responsibility that Japan should undertake when its international unlawful act causes transnational personal, asset, and environmental damage. According to Japanese domestic laws and Article 44 of *the Law of the People's Republic of China on Choice of Law for Foreign-related Civil Relationships*, Chinese victims have the right to launch lawsuits to claim infringement damage compensations in Japanese or Chinese courts. Besides, Japanese and Chinese domestic laws can be used as the proper laws for hearing disputes over compensation for environmental damage caused by Fukushima wastewater discharge.

### 4.1 Legal basis from Chinese domestic laws

#### 4.1.1 The Civil Code of the People’s Republic of China (Civil Code, PRC)

Article 1229 of the law stipulates “where any harm is caused to another person by environmental pollution or ecological damage, the tortfeasor shall assume the tort liability”. If the discharge of Fukushima nuclear wastewater causes damage to people in China, the infringed party has the right to affix the infringement responsibility of the infringing party. Article1232 of the law stipulates “*where a tortfeasor violates the provisions of laws and intentionally causes environmental pollution or ecological damage, resulting in serious consequences, the victim shall have the right to claim corresponding punitive compensation”*. Hence victims of the Fukushima nuclear wastewater pollution have the right to investigate and affix the tort liability and demand compensation liability of the infringing party based on the *Civil Code PRC*.

#### 4.1.2 Law of the People's Republic of China on prevention and control of radioactive pollution

Article 47 of the law stipulates “*it is prohibited to import radioactive wastes or radioactively polluted articles into the territory of the People's Republic of China or to transfer them via the territory of the People's Republic of China*”. When Japan discharges Fukushima nuclear wastewater into the waters under China's jurisdiction, it shall comply with the discharge standards and regulations on discharge approaches proposed by China.

#### 4.1.3 Regulation on the safety management of radioactive waste, PRC

Article 4 of this regulation stipulates that the procedures for the storage and disposal of nuclear wastewater shall comply with “*the principle of reduction, harmlessness, appropriate disposal and permanent safety*”. When the radioactive waste liquid fails to reach the standard for purification treatment, it should be converted into solid waste and processed by units with disposal licenses. Article 34 of this regulation stipulates that it is prohibited to bring radioactive wastes and radioactive-contaminated objects into the territory of the People's Republic of China.

#### 4.1.4 Nuclear safety law, PRC

Article 41 of this law stipulates that “*a nuclear facility operating entity or a radioactive waste processing and disposal entity shall process or dispose of radioactive waste in a way to reduce its quantity and make it harmless in order to ensure permanent safety*”. Article 90 of the law stipulates that “*the nuclear facility operating entity which causes personal injury or death, loss of property or environmental damage by reason of a nuclear accident shall be bound to make compensation according to the national nuclear damage liability system unless it is able to prove that the damage results from such circumstances as war, armed conflict and riot*”. Japan's discharge of nuclear wastewater is an act to dispose of nuclear wastewater without the permission of relevant competent authorities. When Fukushima nuclear wastewater causes pollution in China's territory, China can investigate and affix the legal responsibility of Japan.

### 4.2 Legal basis from Japanese domestic laws

#### 4.2.1 Japan atomic energy basic act

Article 2 of this law stipulates that “*the research, development and utilization of nuclear energy is limited to peaceful purposes, is to aim at ensuring safety*”. Nevertheless, safety supervision was lacking, and relevant emergency measures were not in place when Fukushima Nuclear Plant developed its nuclear energy. Moreover, the plant failed to protect its nuclear facilities. Article 20 of the law stipulates that “*in order to prevent radiation hazards and ensure public safety, the regulations on the manufacture, sale, use, measurement, etc. and any other safety and health measures relating to radioactive materials and radiation generating devices are provided separately by law*”. After an accident occurred, nuclear wastewater was stored in the unique-textured water conservation tank of the Fukushima Nuclear Plant. When the government searched for a proper disposal plan for nuclear wastewater, hundreds of tons of wastewater leaked due to Japan's Tokyo Electric Power Company (TEPCO) 's neglect of management, aging facilities, and workers' failure to operate in strict accordance with standards. Thus, Japan should undertake responsibility for its domestic nuclear security.

#### 4.2.2 Japan act on special measures concerning nuclear emergency preparedness

Article 3 of this act stipulates that “*a nuclear operator shall be responsible for taking full-scale measures for the prevention of the occurrence of a nuclear disaster pursuant to the provisions of this Act or any other relevant Act and for taking, in good faith, necessary measures with regard to the prevention of the progression (expansion) of a nuclear disaster (including the probability of the occurrence of a nuclear disaster) and nuclear disaster recovery efforts*”. Nevertheless, Japan has deviated from the purpose of this act and failed to fulfill its obligation to prevent nuclear disasters in advance and provide emergency information timely afterward, which may severely threaten the public's lives and health. The Government of Japan can be liable for the nuclear damage if it failed to exercise its regulatory power over the Tokyo Electric Power Company or if its errant acts expanded the damage [15].

#### 4.2.3 Japan act on compensation for nuclear damage

This act serves as the basic compensation system for nuclear damage resulting from nuclear accidents. Those harmed by the nuclear accident, as distinguished from those whose injuries were caused by the earthquake or tsunami, are eligible for compensation under legislation that governs the operation of nuclear power facilities [16]. Section 3 of this Act stipulates that “*where nuclear damage is caused as a result of reactor operation etc. during such operation, the nuclearoperator who is engaged in the reactor operation etc. on this occasion shall be liable for the damage, except in the case where the damage is caused by a grave natural disaster of an exceptional character or by an insurrection*”. TEPCO should undertake the liability for compensation according to the *Act on Compensation for Nuclear Damage,* and the Japanese government should undertake the liability for compensation according to the *Japan State Compensation Law*. Thus, the Court of First Instance affirmed the claim for damage compensation. Japan Act on Compensation for Nuclear Damage provides that the operator may be exempted from liability when " … the damage is caused by a grave natural disaster of an exceptional character … ". Where this exoneration applies, the government shall take, pursuant to the Compensation Act, “the necessary measures to relieve victims and to prevent the damage from spreading” [17].

### 4.3 Legal basis from international law

Currently, international conventions on civil liabilities include the *Paris Convention on Third Party Liability in the Field of Nuclear Energy (1960)*, the *Vienna Convention on Civil Liability for Nuclear Damage (1963)*, etc. Many developed countries that develop nuclear energy, including Japan, haven't acceded to the conventions mentioned above because their domestic laws stipulate that domestic victims will accept higher compensation. However, Japan is a signatory of the Convention on Nuclear Safety that was adopted by the IAEA in Vienna, 1994. The IAEA has strengthened its regulatory powers in 1994 with the Convention on Nuclear Safety that establishes standards for member states, but it cannot verify whether the standards are being met nor penalize nations for failure to comply [18]. International environmental laws have proposed a series of principles that require all countries to bear legal responsibilities when their acts run against international obligations and cause transboundary damage to people's lives, properties, and the environment.

Typical examples include the principles that ‘polluters pay’ and ‘the responsibility falls on the country’. Trail Smelter Arbitration case^4^ was the earliest precedent established in cross-border pollution and a transboundary case involving the federal governments of Canada and the United States. Eventually, it contributed to establishing the harm principle and polluter pays principle in cross-border pollution. The tribunal declared that ‘no state has the right to use or permit the use of its territory in such a manner as to cause injury by fumes in or to the territory of another or the properties or persons therein, when the case is of serous consequence’. In other words, a country can pollute its territory as long as it adheres to its domestic laws. If the pollution crosses national borders and results in severe consequences, the country will infringe upon other countries' sovereignty and shall compensate affected countries according to the harm principle. The Lac Lanoux Arbitration case^5^ emphasized the degree and results of pollution. The Tribunal in Lac Lanoux Arbitration case decided that in carrying out, without prior agreement between the two Governments, works for the utilization of the waters of Lake Lanoux in the conditions mentioned in the Scheme for the Utilization of the Waters of Lake Lanoux, the French Government was not committing a breach of the provisions of the Treaty of Bayonne of May 26, 1866, and the Additional Act of the same date. Following the Trial Smelter Arbitration's precedent, the Tribunal thought France's engineering plan didn't severely infringe upon the environmental right of neighboring Spain. Moreover, it fulfilled the notification obligation and didn't undertake legal responsibility. If Japan is to bear the legal responsibility for discharging nuclear wastewater into the sea, countries involved should provide proof that the discharge act has caused significant damage to neighboring countries and investigate whether the Japanese government has fulfilled its notification obligation.

The requirements of these principles can be seen in the International Law Commission of the UN: it is important, as the preamble records of Draft articles on the Prevention of Transboundary Harm from Hazardous Activities, that those who suffer harm or loss as a result of such incidents involving hazardous activities are not left to carry those losses and are able to obtain prompt and adequate compensation [19]. That is to say, a country's legal obligation to compensate for environmental damage resulting from certain activities has been repeatedly confirmed by the international community and constitutes an integral part of international customary laws.

## 5 China's right-safeguarding strategies on the international level

### 5.1 China safeguards its rights through negotiations with Japan

In modern international relations, the merit of bilateral cooperation lies in its high flexibility and the ability to meet both parties' interests to the maximum extent and help both countries' expectations converge in handling international environmental disputes. It's impossible for a country not to interact with the outside world in modern international relations. By observing the principle of “safeguarding sovereignty and considering downstream interests”, China effectively solved issues concerning international waterways through bilateral cooperation in history. The bilateral marine cooperation in Chinese history mainly focuses on signing intergovernmental agreements on scientific and technological cooperation and memorandums of understanding on maritime scientific and technological cooperation. China has signed an intergovernmental scientific and technological cooperation agreement with the United States, France, Japan, and many other countries. If Japan continues to act arbitrarily, despite warnings from various countries, China might consult with South Korea, Russia, and East Asian neighbors, as well as the Pacific Rim fisheries industry chain, to develop a sanctions mechanism that can have a greater impact on Japan and restrict or ban the import of Japanese fisheries products [7].

In the Mox Plant case,^6^ ITLOS established the cooperation principle and the principle of exchanging further information on cross-border marine pollution. ITLOS stated that Ireland and the United Kingdom shall cooperate and shall, for this purpose, enter into consultations forthwith in order to: exchange further information with regard to possible consequences for the Irish Sea arising out of the commissioning of the MOX plant; monitor risks or the effects of the operation of the MOX plant for the Irish Sea; and devise, as appropriate, measures to prevent pollution of the marine environment which might result from the operation of the MOX plant. This precedent pointed out the direction of cross-border pollution issues concerning Japan's discharge of nuclear wastewater into the sea. Japan should cooperate wholeheartedly with vast countries along the Pacific rim, including China, and report and disclose relevant information on time.

China and Japan can conduct bilateral consultations or sign bilateral agreements based on reaching a consensus on environmental standards and common interests. Besides, China and Japan should conduct negotiations through four principles: equality & mutual benefit, voluntary cooperation, mutual accommodation & mutual understanding, and consensus. Before a damaging act is committed, both parties should enhance their technical cooperation and exchange of information to achieve win-win results concerning protecting the ecological environment. After the damaging act is committed, China and Japan can discuss the compensation sum for the transboundary environmental damage caused by Japan's Fukushima nuclear wastewater pollution incident and negotiate how Japan should fulfill its compensation obligation and makes relevant commitments, so as to eventually safeguard China's right to claiming for compensations. In a word, measures should be taken to achieve peaceful coexistence and avoid making the environmental issue political.

### 5.2 China and other countries jointly safeguard their rights

Ahn said joint expressions of opposition in the region could force the Japanese government to choose a safe method to deal with the wastewater instead of dumping it into the sea [20]. Transboundary environmental damage affects a complex ecosystem. Due to the interactions and impacts between each sphere in nature, more than one country is damaged. Thus, all countries should cooperate with concerted efforts to prevent or reduce the impacts of transboundary damage. Region or subregional system arrangements are the most direct manifestations of international environmental commitments. Compared with the grave difficulties of formulating and implementing global agreements, the formulation and implementation of regional agreements face fewer obstacles. While it is difficult for Asian countries to conclude a regional treaty, Japan and its neighboring countries should make diplomatic efforts to shape a soft law that includes cooperative duties, and to implement the hortatory language from the non-binding instruments into obligatory language in the treaty [21]. UNEP (United Environment Programme) has set up an office within the United Nations Economic and Social Council to coordinate regional environmental activities, which serves as a vital guide for countries to conduct regional cooperation. Most of the time, regional rules are basic conditions for formulating global environmental rules and facilitate their development. The measures taken by countries to cooperate in safeguarding their rights are listed as follows:

Firstly, conclude regional framework conventions: As countries damaged by the nuclear wastewater discharge, they have common interest appeals. Thus China can cooperate with other countries that may be damaged by the nuclear wastewater in concluding regional framework conventions. Meanwhile, specific protocols in certain realms can be reached according to each country's economic conditions or political interests to cooperate in capital, technology, research, and personnel exchange.

Secondly, share the monitoring information on the marine environment. The science and technology department of each country should monitor the domestic country's pollution level and share information and data within the framework of regional cooperation. It's conducive to forming a holistic understanding of marine pollution conditions and reaching a consensus on specific environmental pollution prevention and cooperation projects.

Thirdly, establish a compensation fund for transboundary damage. Nuclear pollution lasts for a long time, and its damage can hardly be predicted. As a result, the compensation for transboundary damage can hardly reach the requirements for timeliness and effectiveness. Thus the compensation fund for transboundary damage is established to compensate victims of transboundary pollution acts more efficiently. For example, the *International Convention on the Establishment of an International Fund for Compensation for Oil Pollution Damage* has set up a public compensation fund and proposed specific conditions for compensations to compensate for the property loss and environmental damage caused by transboundary activities to victims.

### 5.3 China safeguards its rights through international institutions

Since its establishment, UNEP has been a major tool for bringing environmental experts together to share experience and jointly solve environmental problems. In many successful cases concerning regional environmental cooperation, UNEP has served as an external impetus, and its regional marine projects are the best examples. Firstly, UNEP can enhance the responsibility, effectiveness, and influence of international relief actions for nuclear wastewater based on the *Global Programme of Action for the Protection of the Marine Environment from Land-based Activities (GPA)* [22]*.* Secondly, countries should coordinate the specific matters and rules applicability under the leadership of UNEP to reduce barriers to each other party in international cooperation and relief actions.

The head of a taskforce from the IAEA said Friday it is examining whether Japan's planned release into the sea of treated radioactive water from the wrecked Fukushima nuclear plant meets international standards, but the decision on whether to go ahead with the plan is up to the Japanese government [23]. China should actively engage in the IAEA's technical working group. Simultaneously, China could join international organizations and Pacific Rim nations in urging Japan to cease releasing nuclear waste and instead pursue alternative viable solutions [7]. China can set up an information-sharing mechanism with IAEA's technical expertise and knowledge and require Japan to disclose information on Fukushima nuclear wastewater in the principle of openness and transparency. On this basis, China needs to negotiate with Japan under the chairing and organization of IAEA, striving to handle Fukushima nuclear wastewater with minimal environmental impacts.

### 5.4 China employs international dispute settlement mechanisms

#### 5.4.1 International arbitration

Flexibility and a certain degree of compulsiveness are two distinctive features of international arbitration. The parties involved can select arbitrators to set up an arbitration court and even choose the laws and procedures applicable to the arbitration. In 2000, the Permanent Court of Arbitration adopted the *Optional Protocol for Environmental Disputes* and made special arbitration rules on environmental disputes in the international community to provide arbitration channels with higher suitability for solving international environmental disputes. Hence these rules are professional in this regard. Since the Alabama Claims, international arbitrations have been adopted by a growing number of countries to solve transboundary damage disputes. Three years later, Western countries were involved in 110 arbitration cases. In international legislation activities, most dispute clauses in international conventions have stipulated how to submit the arbitration and its treatment. The regulations of the *1899 Convention for the Pacific Settlement of International Disputes* and the *1907 Convention for the Pacific Settlement of International Disputes* have affirmed international arbitration practices. It symbolizes that arbitration, a way to resolve international disputes, plays a growingly important role in international laws. International arbitration has emerged as a significant means for resolving environmental disputes in the current international community. Important regional or global environmental conventions have stipulated that arbitration is one of the settlement means. For example, the *Protocol on Environmental Protection to the Antarctic Treaty* claims that as required by any party, the dispute should be submitted for arbitration or resolved in the same approach accepted by both parties.^7^ *Convention* and *Convention on Environmental Impact Assessment in a Transboundary Context* also stipulate that the country involved can submit the arbitration according to procedures stipulated by these conventions.^8^ From the theoretical perspective, arbitration should be conducted based on the agreement of the country involved. It's because international arbitration in the principle of national sovereignty is essentially a voluntary jurisdiction. Therefore, international arbitration is consistent with the common interests of China and Japan, whether from the perspective of flexibility, professionalism, and voluntariness. Besides, it's a feasible backup plan to sign an arbitration agreement with Japan. Meanwhile, international arbitration can expand the channels for China to safeguard national and civil interests, if the damage compensation issues cannot be solved through negotiations and cooperation between China and Japan after the damage occurs.

#### 5.4.2 International justice

Japan declared that it would recognize the jurisdiction of the ICJ since July 9, 2007 [24]. Regarding the similarity, international justice and international arbitration are based on the common consent of both parties. In terms of the difference, the laws and procedural suitability in international justice are more strict and stable. According to the regulations of the *Statute of the International Court of Justice*, the International Court of Justice (ICJ) can govern all cases submitted by the countries involved and all events stipulated by the *UN Charter* or existing conventions and agreements. ICJ is a judiciary institution with universal jurisdiction. Thus international environmental disputes can naturally be submitted to ICJ for settlements. From the perspective of resolving international disputes, the lawsuits accepted by the ICJ involve the explanations and suitability of conventions, territorial sovereignty, and the marine environment. Hence disputes should be resolved by peaceful means to avoid the tension states that may aggravate and threaten peace, thereby safeguarding the legal order of the international community. From the perspective of environmental protection, ICJ has considerably facilitated the development of international environmental laws based on previous judgments and consultation opinions, such as New Zealand v. France Nuclear Tests case,^9^ another case involving nuclear damages in the Pacific. Taking into account both scientific accuracy and social needs [25], the precautionary principle should be applied here and Japan should be required to implement precautionary measures, evaluate the possible impact of nuclear-contaminated water discharge activities on the marine environment, and report the results of the evaluation to competent international organizations such as the agency and States parties [26].

The ICJ in Certain Activities Carried Out by Nicaragua in the Border Area Case^10^ held that the construction of road by Costa Rica carried a risk of significant transboundary harm and that Costa Rica therefore had an obligation under general international law to carry out an environmental impact assessment. However, Nicaragua had not proved that Costa Rica's conduct caused significant transboundary harm and it therefore dismissed Nicaragua's claims on this point. Meantime, the court concluded a declaration of wrongful conduct in respect to Costa Rica's violation of the obligation to conduct an environmental impact assessment. According to the Certain Activities Carried Out by Nicaragua in the Border Area Case, environmental impact assessment and transboundary harm are equally important grounds for accountability in Japan's discharge of Fukushima nuclear wastewater. Furthermore, environmental assessment is even more important in the order of procedure. If the Japanese government doesn't make any environmental impact assessment, the discharge of nuclear wastewater will violate the precedent case created by ICJ. Although China didn't participate in the cases governed by international justice as a country involved, it took part in the consulting procedures for ICJ's case of Kosov's declaration of independence unilaterally.

Moreover, the judgments made by ICJ are legally binding and must be implemented by relevant countries. Thus, the Security Council has the right to propose suggestions on implementing a judgment when relevant countries refuse to implement it. Facing Japan's discharge of Fukushima nuclear wastewater into the sea, China should actively participate in the consulting procedures for the nuclear wastewater dispute cases between Japan and other countries, offer support for safeguarding the rights of countries damaged by the nuclear wastewater, and state its stances on the international platform.

### 5.4.3 International tribunal for the law of the sea (ITLOS）

ITLOS is a special court set up to adapt to the new situation of international environmental protection in accordance with provisions of the *Statute of the ITLOS (Annex VI of the UNCLOS)*. It can resolve the disputes caused by the explanations and suitability of each country for *UNCLOS*. ITLOS has broken away from the limitations of traditional international laws. While ICJ only accepts cases where the parties involved are countries, ITLOS can accept cases with subjects other than countries. Therefore, ITLOS is more applicable to the characteristics of current environmental disputes and more professional when resolving national environmental disputes. Aside from public prosecution for the dispute over Fukushima nuclear wastewater discharge, international organizations or partial enterprises, as subjects of lawsuits, can actively safeguard environmental rights and jointly govern the international environment. In addition, Article 290 of *UNCLOS* stipulates that *provisional measures, as special procedures for resolving marine environmental disputes, also play a critical role in ITLOS's practices.* For example, Southern Bluefin Tuna Case and MOX Plant Case (Ireland v. United Kingdom) are both marine environmental dispute cases where temporary measures apply. Thus, China can apply temporary measures to ITLOS by taking environmental factors as primary appeals, adopting preventive measures, and taking specific actions when necessary to prevent environmental deterioration.

## 6 China's right-safeguarding strategy on the domestic law

### 6.1 Retention of proof

To prove the causality between the pollution in China's waters and Japan's discharge of nuclear wastewater, the damaged party should have a high technical and economical level. If individuals or enterprises seek relief as lawsuit subjects, they generally fail to collect or provide relevant proof, owing to inadequate technical and economical competence. Therefore, the country should set up a professional radioactive pollution evaluation mechanism to safeguard nationals' lawful interests, test the level of radioactive elements in ocean creatures within China's waters, and track the changes in the radioactive values of seawater and the ocean's ecological functions. The State Oceanic Administration stated in 2011 that relevant departments are advised to enhance the testing of the radioactivity of marine products in these waters to safeguard the Chinese people's health and security [27]. Therefore, China should enhance the testing of water and creature species that can be used as evidence to reduce the difficulty for individuals or enterprises to propose proof. The data published by the national technical department are authoritative and can be easily accepted as proof. Concrete and feasible measures are listed as follows:

To raise the monitoring frequency and widen the monitoring scope. The monitoring samples of different monitoring stations at different periods should be retained to analyze the dynamic changes in radioactive materials. Considering Japan's plan to discharge nuclear wastewater into the sea is proceeding, the frequency for monitoring should be raised accordingly to ensure each change in the radioactivity value is recorded. Meanwhile, the provided data should be accurate, and all evidence should be real. On a scientific basis, the monitoring scope can be expanded to the waters without human activity but should receive due attention. In this way, preventive measures can be adopted once any abnormality occurs in the radioactivity value of these waters to avoid nuclear pollution in the waters with human activities. China could join forces with Pacific Rim nations, particularly South Korea, ASEAN countries, Australia, and New Zealand, to send experts to Japan to sample and inspect non-discharged wastewater to determine the extent of the risks [7].

To enhance the accuracy of monitoring data. Firstly, representative seawater samples should be collected from different regions and monitored in strict accordance with standard methods. Meanwhile, samples should be prepared based on retaining their original characteristics, making it convenient to compare them with samples collected from different waters or at a different monitoring time. Secondly, testing and other equipment should operate stably to eliminate interference factors in the monitoring process to the maximum extent and guarantee the monitoring results' accuracy. Besides, the radioactive amount of seawater and diverse ocean creatures should be monitored at any time and updated timely to provide evidence for claiming compensation. Lastly, the monitoring data should be processed scientifically to enhance their accuracy.

### 6.2 Confirmation of China's civil jurisdiction

Jurisdiction and the choice of laws are equally important in rules on jurisdiction conflicts. For the infringement disputes in transboundary damage compensation cases, an emphasis should be laid on the private interest nature of infringements, and protecting victims is the center and starting point. However, the 2006 Act *on General Rules of Application of Laws in Japan* stipulates that applied laws should observe the model of overlapping and suitability in environmental infringements.^11^ That is to say, foreign laws' applicability is restricted when overlapping the local laws of infringement acts and the local laws of the court. As a result, the procedures for handling the case are complicated, and the effectiveness of receiving relief is too long, which is unfavorable to Chinese victims. Therefore, affirming that China enjoys civil jurisdiction in the transboundary damage cases of nuclear wastewater discharge is critical for giving full play to domestic relief means. The party involved's nationality, residence, habitual residence, place of act occurrence, and the place of launching the lawsuit are all factors worth considering when exercising the right of jurisdiction. In the lawsuits for transboundary environmental damage compensations, the international community mainly considers the place of act occurrence and the place of damage because these factors are more substantially connected to the occurrence of transboundary damage.

After Japan discharges Fukushima nuclear wastewater into the sea and pollutes the waters within the jurisdiction of China, the regulations of the *Draft Articles on the Prevention of Transboundary Harm from Hazardous Activities* stipulate that Japan, as the country where the damage originates, and China, the country which experiences the transboundary environmental damage, both have the right of jurisdiction in this transboundary damage case. If actual pollution and damage are caused after Japan discharges Fukushima nuclear wastewater into the sea, the individuals or enterprises damaged in China have the right to launch lawsuits to claim compensation for the pollution damage to the domestic courts with the jurisdiction right [28]. Besides, the victims have the right to claim compensation according to Chinese domestic laws applicable to this case. It avoids the disadvantage that applying foreign legal procedures is too complicated and fails to provide judicial remedies in time.

### 6.3 China's right to national subrogation

Protecting its nationals is a country's right and obligation. *The Constitution of the People's Republic of China* protects and respects citizens' fundamental rights.^12^ The nationals mentioned here do not refer to an abstract whole, but involve every specific person. The *Draft Articles on the Prevention of Transboundary Harm from Hazardous Activities* stipulates that victims should receive timely, adequate, and effective relief.^13^ Nevertheless, previous cases concerning compensations for transboundary environmental damage involve national interests, so it generally lasts for several years, from the proposal of a case to demonstrating case facts and eventually judging the case. Take Trail Smelter Arbitration^14^ as an example. Both countries involved signed an arbitration agreement on April 14, 1935. But it wasn't until 1941 did the arbitration court make the final judgment. Considering the regional uniqueness of transboundary, ordinary victims can hardly bear heavy time and economical costs. To guarantee timeliness, sufficiency, and effectiveness, the country should first pay damage compensations to victims in advance and get the right to subrogation. Then the country can propose a claim for compensation to the country of source. This is an effective means for protecting nationals' obligations. Nevertheless, it should be noted that a country's right to subrogation is not about offering relief directly but about providing economic relief after the compensation judgment is made through judicial means, yet victims are still waiting for its execution.

In the lawsuit of compensation for transboundary damage resulting from discharging nuclear wastewater into the sea, it is advisable to set up a special domestic fund through national taxation and the environmental performance bonds, wastewater discharge fees, and environmental fines paid by enterprises to guarantee the country's right to subrogation and serve as its fund guarantees [29]. Meanwhile, special procedures should be proposed to set up a legal subrogation system from procedures to objects and from affirmation to implementation. This system ensures that transboundary damage compensations can be effectively guaranteed through domestic relief means, which avoids high costs for seeking judiciary relief in transboundary damage compensation cases.

## 7 Conclusions

Although the United Nations Convention on the Law of the Sea, the Convention on Nuclear Safety, and the Convention on Early Notification of a Nuclear Accident do not explicitly oppose discharging nuclear waste into the sea, international treaties such as the United Nations Convention on the Law of the Sea stipulate the obligations of the parties to accidents that may cause transboundary effects [7]. The Japanese government's decision to discharge Fukushima nuclear wastewater into the sea and its future discharge act are suspected of violating international laws. As a contracting country of the *Convention on Nuclear Safety*, the *London Convention*, the *Convention on Early Notification of a Nuclear Accident*, and *UNCLOS*, Japan willfully decided to discharge Fukushima nuclear wastewater, which fails to reach ALPS treatment standards, into the Pacific Ocean without fulfilling the obligations for transboundary environmental assessment, notification, and negotiations. In this case, Japan should bear the unshakable national and civil liabilities for its discharge act. Regarding Japan's act to discharge the nuclear wastewater, China should facilitate the international community's adherence to the concept of building a community of a shared future for mankind, improve the legal system for international nuclear security, enhance the monitoring of diverse subjects, and urge Japan to comply with international laws. More specifically, China should handle nuclear pollution with methods on two levels: On the international level, China should sign bilateral agreements, enhance regional collaboration, and reasonably utilize international dispute settlement mechanisms, etc. On the domestic level, China should focus on remedies of domestic laws, including exercising international civil jurisdiction, claiming remedies, and actively exercising the right of subrogation of the state. These measures are not only the bottom lines for protecting victims' rights and interests in judicial remedies, but also constitute a critical component of the transboundary damage liability system.

## Author contribution statement

All authors listed have significantly contributed to the development and the writing of this article.

## Data availability statement

Data included in article/supp. material/referenced in article.

## Additional information

No additional information is available for this paper.

## Declaration of interests

The authors declare the following financial interests/personal relationships which may be considered as potential competing interests.

Meng Li reports financial support was provided by “Youth Innovation Team Plan” of Shandong Provincial Universities approved by 10.13039/100012906Shandong Provincial Education Department in 2022 (Grant No. 2022RW016): “Research on anti-terrorism legislation and Human Rights Protection”.

Xuedong Wang reports financial support was provided by 10.13039/501100018563Social Science Planning Research Project of Shandong Province in 2022 (Grant No. 22CFXJ13): “Study on the legal Guarantee of eco-environment collaborative governance in the Yellow River Basin”.

Meng Li reports financial support was provided by Innovation Scientific Research Plan Project (Young People's Scientific Research Start-up Fund) approved by 10.13039/501100002862China University of Petroleum (East China) in 2022 (Grant No. 22CX06075A/27RB2211012): “Studies of the Public Interest in Judicial Review──from the perspective of British Common Law, EU Law and International Law”.

## Footnotes

1 See Pulp Mills on the River Uruguay (Argentina v. Uruguay), Judgment, I.C.J. Reports 2010.

2 See Southern Bluefin Tuna Case (Australia v. Japan), Provisional Measures, ITLOS Nos. 3&4, Order of 27 August 1999.

3 See Principle 15 of the Rio Declaration: Where there are threats of serious or irreversible damage, lack of full scientific certainty shall not be used as a reason for postponing cost-effective measures to prevent environmental degradation.

4 See The Trail Smelter Arbitration (the U.S. v. Canada) (1941), 3 RIAA 1905.

5 See The Lac Lanoux Arbitration (Spain v. France) (1957), 24 I.L.R.

6 See MOX Plant (Ireland v. the United Kingdom), Provisional Measures, Order of 3 December 2001, ITLOS Reports 2001.

7 See Article 18 of Protocol on Environmental Protection to the Antarctic Treaty: If a dispute arises concerning the interpretation or application of this Protocol, the parties to the dispute shall, at the request of any one of them, consult among themselves as soon as possible with a view to having the dispute resolved by negotiation, inquiry, mediation, conciliation, arbitration, judicial settlement or other peaceful means to which the parties to the dispute agree.

8 See Appendix of Convention and Convention on Environmental Impact Assessment in a Transboundary Context 1991: The claimant Party or Parties shall notify the secretariat that the Parties have agreed to submit the dispute to arbitration pursuant to Article 15, paragraph 2, of this Convention.

9 See Nuclear Test Cases (Australia v. France, New Zealand v. France), December 20, 1974. International Court of Justice, 9 INT'L L. 563 (1975).

10 See Certain Activities Carried Out by Nicaragua in the Border Area (Costa Rica v.Nicaragua) and Construction of a Road in Costa Rica along the San Juan River (Nicaragua v. Costa Rica), Judgment, I.C.J. Reports 2015.

11 See Article 22 of 2006 Act on General Rules of Application of Laws in Japan: (1) In the case where a tort is governed by a foreign law, if facts to which the foreign law should be applied do not constitute a tort under Japanese law, no claim under the foreign law may be made for compensation or any other dispositions. (2) In the case where a tort is governed by a foreign law, even if facts to which the foreign law should be applied constitute a tort both under the foreign law and Japanese law, the victim may make a claim only for compensation or any other dispositions that may be permitted under Japanese law.

12 See Chapter 2 of China's Constitution stipulates the fundamental rights of Chinese citizens.

13 See Article 3 (10): In the context of the present articles, due diligence is manifested in reasonable efforts by a State to inform itself of factual and legal components that relate foreseeably to a contemplated procedure and to take appropriate measures, in timely fashion, to address them. Thus, States are under an obligation to take unilateral measures to prevent significant transboundary harm or at any event to minimize the risk thereof arising out of activities within the scope of article 1.

14 See Trail Smelter Arbitration (United States v. Canada) (1938 and 1941) 3 R.I.A.A. 1905.
